# *Salmonella* isolated from street foods and environment of an urban park: A whole genome sequencing approach

**DOI:** 10.1371/journal.pone.0320735

**Published:** 2025-04-02

**Authors:** Christian Vinueza-Burgos, José Medina-Santana, Maria Ishida, Brian Sauders, Gregory Deiulio, Alyssa Dickey, Pablo Endara, Rommy Terán

**Affiliations:** 1 Unidad de Investigación de Enfermedades Transmitidas por Alimentos y Resistencia a los Antimicrobianos (UNIETAR), Facultad de Medicina Veterinaria y Zootecnia, Universidad Central del Ecuador, Quito, Ecuador; 2 Division of Food Laboratory, New York State Department of Agriculture and Markets, Albany, New York, United States of America.; 3 Escuela de Medicina, Colegio de Ciencias de la Salud, Universidad San Francisco de Quito, Quito, Ecuador; 4 Facultad de Ciencias Químicas, Universidad Central del Ecuador, Quito, Ecuador; University of Tripoli, LIBYA

## Abstract

*Salmonella* is one of the most important foodborne pathogens worldwide. Therefore, this study was conducted to understand the importance of this microorganism in street food and the environment of an urban park in Quito, Ecuador. This research included phenotypic characterization and whole genome sequencing (WGS) analysis of isolates from different food matrices and fecal samples of dogs and pigeons. *Salmonella* was found in 10% (18/180) of the food samples, 3% (3/100) of the dog stool samples, and 5% (5/100) of the pigeon stool samples. These results also showed that meals containing any sauce or eggs were associated with a high probability of *Salmonella* isolation, regardless of other ingredients. All *Salmonella* isolates from food were identified as *Salmonella enterica* serovar Typhimurium (*S.* Typhimurium) while isolates from animal feces belonged to *Salmonella enterica* serovar Infantis (*S.* Infantis) and *S.* Typhimurium. WGS analysis showed that all *S.* Typhimurium strains belonged to ST19 and *S*. Infantis to ST32 according to the Multi-Locus Sequence Type (MLST) scheme. These strains were not related to *Salmonella* genomes of other origins when a Single Nucleotide Polymorphism (SNP) tree analysis was carried out. Antimicrobial resistance genes, such as *bla*_CTX-M-65_, were predominantly linked to the pESI-like plasmid found in *S*. Infantis. These results show the importance of urban fauna as a reservoir of *S.* Infantis and the impact these animals could have in terms of public health.

## Introduction

Meals served and sometimes cooked in public places are named street food. These foods are widely exposed to potential microbial contamination through soil, dust, and water contact. Besides, preparation methods, holding temperature, and food-handlers’ sanitary practices are factors related to the risk of bacterial contamination [1].

Pathogenic microorganisms like *Salmonella* have been reported in street food [2–4], causing food-borne illnesses. This pathogen can cause diseases ranging from diarrhea to septicemia according to the serotype [5]. Besides, the recurrent reports of resistant strains of this pathogen lead to problems related to the disease treatment [6–8]. Therefore, isolation and characterization of *Salmonella* in different food matrices are essential in the active surveillance of safety and quality control of food [6].

Millions of low-and-middle-income consumers eat street food regularly in developing countries, mainly due to the low cost and easy access to these foods. Additionally, the growth of street food vendors promoted by socioeconomic conditions (migratory crisis, inflation, etc.) has raised the risks of food poisoning and outbreaks [1]. These small businesses are usually located in urban parks where urban animals are present, especially dogs and pigeons [2,3]. Dogs play many roles such as pets, guarding, lifesaving, and they are important in the physical and psychological human well-being. Nonetheless, dogs may represent a potential risk for human health due to the possibility of the transmission of zoonoses [4]. Thus, dogs can be infected, even asymptomatically, by several zoonotic enteric bacteria (species of *Salmonella*, *Campylobacter*, *Yersinia*, *Listeria*, etc.) that later are excreted and spread by their feces, representing a risk of infection to the human population [9].

On the other hand, populations of feral pigeons are dense in cities and could act as reservoirs and vectors of diseases to other birds and humans. Pigeons, due to their tendency to forage in garbage and animal remains, play a significant role in the spread and epidemiology of certain bacterial pathogens [5]. *Salmonella* is among the primary zoonotic bacteria reported to be harbored by pigeons[10] which potentially implicates these birds in the transmission of salmonellosis in urban environments [6].

When dog feces are not regularly removed from public places such as parks, it might represent a risk factor because organic matter can be spread by wind, vehicular and bike traffic, contaminating food and water [7]. Similarly, pigeons can be considered a risk factor when contaminated feces reach food and water after traveling several kilometers [8]. Consequently, microbiological surveillance of street food should be implemented as a strategy to reduce the risk of foodborne diseases. Plasmids play a crucial role in bacterial evolution, conferring antimicrobial resistance and enhancing host adaptation. Initially, plasmids were classified into Incompatibility (Inc) groups based on coexistence capacity within a cell [11], yet this method lacks insight into genetic variants and phylogenetic relationships. With this background, a sequence-based typing scheme named plasmid multilocus sequence typing (pMLST) has been proposed [12]. Moreover, plasmids exhibit genetic plasticity, altering gene order without affecting function, and complicating epidemiological research. Thus, combining Inc groups with pMLST enhances our grasp of plasmid dynamics in bacterial communities. Thus, the present study aims to evaluate the occurrence and genetic profiles of *Salmonella* in the food cooked and commercialized in the biggest urban park of Quito city. This study also evaluates whether dog’s and pigeon’s feces can be contaminated with this bacterium and be a potential source of infection for humans.

## Materials and methods

### Study area and design

This cross-sectional study was conducted from July to September 2018 in Carolina Park, situated in Quito-Ecuador. Quito is the capital of the country, and this park is the largest one in the city (61.4 ha; 0º11’019” S -78º29’04.9” W) nestled in a bustling area surrounded by schools, office buildings, and shopping centers (S1 file). More than 100.000 people visit it every week and is particularly known for its dog-friendly environment, numerous pets, and stray dogs living in the park. Besides, this green area is the habitat of many wild and urban birds including pigeons. Contamination from dogs and pigeon stools is commonly seen in several areas. This park is also a place where people prepare and sell street foods, providing visitors with a variety of local snacks and meals. However, there is a lack of clean water and adequate food safety measures.

### Sample collection

Sample size was defined by convenience because there is no valid information about the dogs and pigeons’ numbers in this location, nor about the numbers of food sellers and prevalence of *Salmonella* in street foods. Moreover, this study was not intended to measure the prevalence of *Salmonella* in street foods, but to establish the genomic relationship among *Salmonella* isolates.

The park of study has six locations (Children’s play area, Lagoon Area, Botanical garden, Running track, Skating area, and sports facilities). Vendors occupied these areas without established regulations. Five different vendors were selected in each area and 3 food samples were acquired from each vendor. So, a total of 15 food samples from each area were collected. This procedure was repeated 7 days later in the same locations but with different vendors reaching 180 food samples for final analyses. Foods were categorized according to their susceptibility to contamination core ingredients. Core ingredients do not necessarily mean the main ingredient, but those that, due to handling or limited cooking processing, imply a greater risk of contamination for the dish. These categorized ingredients were: sauce, fruit, beef, chicken, eggs, sausages, milk, potatoes, and chocho (pearl lupin). However, several foods shared some core components, and many were composed of a mixture of cooked and raw ingredients (S2 file).

A total of 200 stool samples (100 pigeons and 100 dogs) were collected for 6 Sundays in the same locations as the food vendors. Fecal samples were identified as freshly deposited by visual inspection and subsequently collected as individual samples.

All samples were individually packed in sterile plastic containers, labelled (date of sampling, location, number and type of sample) and transported at 4°C in cooler boxes with ice packs to the microbiology laboratory for processing within 48 hours of collection. Sample collection did not involve live animals nor human samples. Therefore, no permits were needed according to local regulations.

### Microbial analysis of food

The food samples analysis was carried out according to the NTE INEN 1529-15:2009 Ecuadorian standard [9]. Shortly, 25g of the food sample was homogenized in 225 ml peptone water (Conda, Madrid, Spain), and incubated at 37°C overnight. Following enrichment, 1 ml of diluted sample was inoculated in 10 ml tetrathionate broth with brilliant green (Conda, Madrid, Spain) and in 10 ml selenite cystine broth (Merck, Darmstadt, Germany). The incubation temperatures were 42°C and 37°C respectively; both tubes were incubated for 48 hours. Following the second enrichment, a loopful of each broth was inoculated into *Salmonella-Shigella* agar (SSA) (Conda, Madrid, Spain); Bismuth sulfite agar (Conda, Madrid, Spain); Brilliant green phenol red lactose agar (Neogen, Lansing, USA) in duplicates and incubated at 37°C overnight [9]. After incubation, colonies were isolated for further biochemical analyses and stored at -80 °C until antibiotic resistance testing and molecular analysis. American Type Culture Collection (ATCC) strain 14028 was used as a positive control in all processes.

### Microbial analysis of stool samples

Stool samples were analyzed using the ISO 6579:2002 standard [10]. Approximately 1 g of the stool sample was homogenized in 9 ml peptone water (Conda, Madrid, Spain) mixed properly and incubated at 37°C overnight. The 1 ml diluted sample was inoculated in 10 ml Rappaport Vassiliadis broth (Conda, Madrid, Spain) at 42°C overnight. Then, a loopful of broth was inoculated into Xylose Lysine Deoxycholate agar (BD, Sparks, USA) in duplicates and incubated at 37°C overnight [11]. After incubation, suspected colonies were isolated for further biochemical analyses and stored at -80 °C until antibiotic resistance testing and molecular analysis.

### Antimicrobial susceptibility testing

The antimicrobial susceptibility of *Salmonella* isolates was tested by the Kirby-Bauer disc diffusion method following the Clinical and Laboratory Standards Institute (CLSI) guidelines [12]. Antibiotics were selected to represent the major antibiotic groups commonly used in human and veterinary medicine in Ecuador. Tested antibiotic (Oxoid, Basingstoke, UK) were Ertapenem (ETP, 10 μg), Cefoxitin (FOX, 30 μg), Cefotaxime (CTX, 30 μg), Trimethoprim +  Sulfamethoxazole (STX, 1.25/23.75 μg), Streptomycin (S, 10 μg), Nitrofurantoin (F, 300 μg), Amoxicillin +  Clavulanic acid (AMC, 20/10 μg), Chloramphenicol, (C, 30 μg), Gentamicin (CN, 10 μg), Azithromycin (AZM, 15 μg), Fosfomycin (FF, 50 μg), Ciprofloxacin (CIP, 5 μg), Tetracycline (TE, 30 μg), and Amikacin (AK, 30 μg). Cutoff values from CLSI 2022 were used for Kirby-Bauer interpretation [12].

### DNA extraction and whole genome sequencing

Wizard Genomic DNA Purification Kit (Promega, Madison, WI) was used to extract the Genomic DNA according to manufacturer instructions. *Salmonella* isolates were confirmed by PCR according to Akiba, et al. [13]. DNA concentration and quality were measured by Qubit fluorometer system (Invitrogen/Thermo Fisher Scientific, Waltham, MA) and the NanoDrop 2000 UV-Vis (Thermo Fisher Scientific, Wilmington, DE). DNA extracts with concentrations greater than 10 ng/mL and an A260/280 ratio of 1.75 to 2.05 were directly sequenced. However, DNA extracts outside this range could be integrated always that data quality based on coverage (>40X) and other quality assessments inherent in the analysis pipelines were satisfactory. MiSeq platform (Illumina, San Diego, CA) was used to perform whole genome sequencing (WGS) according to the harmonized FDA GenomeTrakr/CDC PulseNet protocol (CDC PulseNet, 2018). Sequences were uploaded to the National Center for Biotechnology Information (NCBI) Sequence Read Archive (SRA). Accession numbers of all genomes studied genes, and their metadata are listed in S3 File.

### Bioinformatic analysis

The integrated software environment EnteroBase was used for quality control, trimming, and assembly of raw reads [14,15]. This platform also functions to identify serotypes, Sequence Type (ST) based on MLST and cgMLST schemes, carrying out SNP tree analysis, and supplying assembled sequences for further analysis. Visualization and annotation of SNP tree analysis were performed on iTOL v. 6.0 [16]. The genomes were compared between them and with other *Salmonella* genomes of different origins (humans, poultry, environment, and wildlife) isolated in Ecuador using sequences available in Enterobase.

Additionally, genes and SNPs that determine resistance to antimicrobials and disinfectants were identified by AMRfinder plus and ResFinder database [17,18]. Virulence genes were identified by the Virulence Factor Database (VFDB)[19] using a mass screening of contigs in ABRicate software [20].

Functional nitrofurantoin resistance-associated mutations were identified as previously described [21]. Briefly, all genomes were mapped to the wild-type sequences of *nfsA* and *nfsB* genes (oxygen insensitive nitro reductase enzymes) of *S. enterica* accession number NC_003197 (Geneious prime v. 2021.0.3). Once sequences were identified, those that possessed mutations were in silico translated and aligned with the wild type proteins sequences to visualize nonsense mutations [22].

In order to identify plasmids, Platon v.1.6 [23–26] was used for plasmid contigs prediction. Additionally, Geneious Prime 2022.1.1. (https://www.geneious.com) and NCBI–BLAST [27] were used to profile the plasmids’ identity. For that, tentative plasmids were mapped in all genomes using the map to reference tool in the Geneious Prime software.

### Statistical analysis

Examined variables were analyzed as qualitative exposures (presence or absence) for meals and nominal for collection place. The relative frequency of each category was expressed in percentage. Multicomponent food was treated according to the main core ingredient in food as stated earlier. Fischer exact test was used to explore the association of each core-ingredient meal and the presence of *Salmonella*. Logistic regression analysis was used to calculate the association (odds ratio) between each meal ingredient and the possibility of *Salmonella* isolation in univariate and multivariate analysis. All analyses were made in STATA software V.15 [28]. Significant results were considered when p-values were equal or under 0.05.

## Results

### Association between specific type of food and *Salmonella* Isolation

From the 380 samples analyzed, twenty-six isolates of *Salmonella* were recovered. Among these isolates, *Salmonella* was found in 10% (18/180) of food samples, 3% (3/100) of dog stool samples, and 5% (5/100) of pigeon stool samples (S3 file). Meals containing sauces, chicken, potatoes, and eggs were associated with higher frequency of *Salmonella* isolation in univariable analysis. After considering the effect of the other ingredients, the only main ingredient associated with higher probability of *Salmonella* isolation were meals containing some sauce or eggs. The presence of sauce or eggs increases 7.9 and 15 times the probability of *Salmonella* isolation respectively (Table 1).

**Table 1:** Uni and multivariate analysis of association between ingredients of street food and *Salmonella* isolation.

Food	Prescence *Salmonella* (%)	P-value (Fisher test)	OR (95%IC)	P-value (OR)	Adj OR	P-value (adj OR)
Sauce	8/18 (44.4)	<0.0001	9.2 (3.1-27.2)	<0.0001	7.9 (2.3-27)	0.001
Fruit	3/18 (16.7)	0.99	1.05 (0.28-3.8)	0.94	3.4 (0.63-18)	0.15
Meat	4/18 (22.2)	0.99	0.9 (0.3-3)	0.91	0.68 (0.11-4.1)	0.68
Sausages	6/18 (33.3)	0.21	2.2 (0.76-6.3)	0.14	1.62 (0.44-5.8)	0.46
Chicken	7/18 (38.9)	0.015	3.8 (1.35-10.9)	0.01	1.9 (0.45-7.7)	0.38
Milk	0/18 (0)	0.13	NA	NA	NA	NA
Potatoes	6/18 (33.3)	0.02	4 (1.33-11.9)	0.01	4.17 (0.79-21)	0.09
Chocho	0/18 (0)	0.37	NA	NA	NA	NA
Eggs	7/18 (38.9)	<0.0001	8.73 (2.8-27)	<0.0001	15 (4.1-55.8)	<0.0001

NA: Not applicable

### *Salmonella* serotypes

All *Salmonella* isolates that originated in food were identified as *S.* Typhimurium while *Salmonella* isolates that originated in animal feces belonged to the serotypes *S.* Infantis and *S.* Typhimurium (Table 2)*.*

**Table 2:** Number of *Salmonella* isolates originated in each sample type.

Samples origin	*S.* Infantis	*S.* Typhimurium	Total
Dog feces	2	1	3
Pigeon feces	5		5
Street food, artisan eggnog		2	2
Street food, barbecue		2	2
Street food, chicken brochette		2	2
Street food, egg foam		4	4
Street food, French fries		2	2
Street food, hamburger		1	1
Street food, hot dog		1	1
Street food, quail eggs		1	1
Street food, roast gizzards		3	3
Total	7	19	26

### Phenotypic and genetic antimicrobial resistance and plasmids identification

All *S.* Typhimurium isolates showed phenotypic resistance to streptomycin and harbored the chromosomal *aac(6’)-Iaa* gene linked to aminoglycoside resistance. These isolates were sensitive to all other tested antibiotics including other aminoglycosides (S3 file).

*S.* Typhimurium strains harbored three different plasmids: pPNCS007087.2 (accession: CP044969), pPNCS014854_S1 (accession: CP037873), and pSe32 (accession: CP067340). Besides, all *S.* Typhimurium strains presented the Incompatibility Group F (IncF) (Sequences IncFIB(S) & IncFII(S)). These strains showed the plasmid Multi Locus Sequence Type (pMLST): [S1:A-:B17].

On the other hand, all *S.* Infantis harbored the plasmids p1864-3 (CP084495) which did not present any resistance genes, and pCFSAN059940 (accession: CP074342) with several resistance genes. The replicon IncFIB(pN55391) was identified in pCFSAN059940-like plasmid that could be integrated into the Incompatibility Group I1 (IncI1). However, the p1864-3 plasmids found in this study were not detected by the PlasmidFinder.

**Fig 1:**
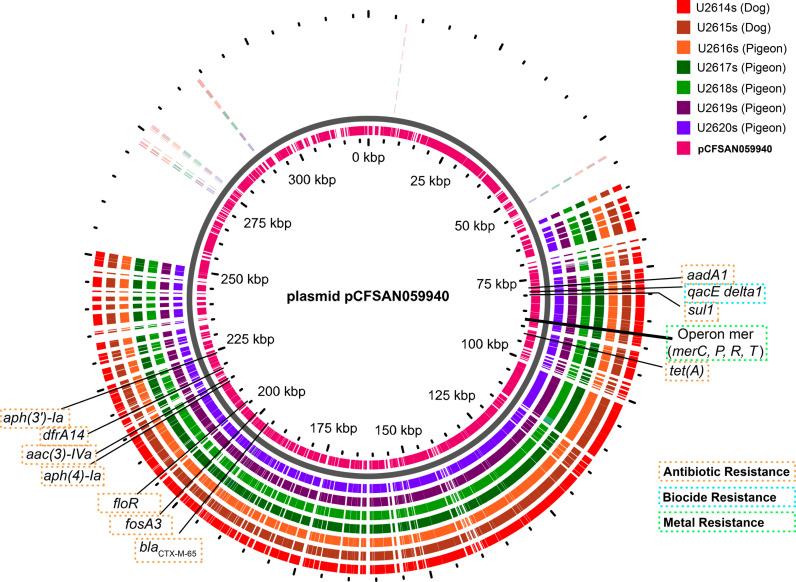
Alignment of pCFSAN059940-like plasmids presents in *S*. Infantis strains with the reference plasmid. The alignment with the reference plasmid, showed a mean coverage of 58.4% and a pairwise identity of 98.1% with entire blocks reorganized or absent in the strains. Inner ring represents the reference plasmid pCFSAN059940.

All *S.* Infantis strains showed the same phenotypic resistance pattern (Table 3). Most of the phenotypic resistance of *S*. Infantis strains was linked to antimicrobial resistance genes harbored in the pCFSAN059940-like plasmid (PESI-like). However, resistance to fosfomycin, sulfamethoxazole +  trimethoprim, and some Beta-lactams did not express phenotypically even though related genes were present (silenced genes) (Table 3). This plasmid was the only one related to antimicrobial-resistance, biocide-resistance, and metal-resistance genes found in the studied strains (Fig 1). The lack of *repI1* locus in pCFSAN059940 plasmids did not allow us to assign them to any known pMLST. However, the nearest sequence was pST 71.

**Table 3:** Antibiotic resistance of *Salmonella* Infantis.

Antibiotic Family	Antibiotic	Number of resistant isolates	Antimicrobial resistance determinant	Related plasmids
Aminoglycosides	Gentamicin	7	*aac(3)-IVa* *aadA1* *aph(3’)-Ia* *aph(4)-Ia* *aac(6’)-Iaa* ^a^	*pCFSAN059940*
	Streptomycin	7		
	Amikacin	0		
Beta-lactams	Cefotaxime	7	*bla*_CTX-M-65_	*pCFSAN059940*
	Ceftazidime	0		
	Cefoxitin	0		
	Amoxicillin ^+ ^ Clavulanic acid	0		
Phenicol	Chloramphenicol	7	*floR*	*pCFSAN059940*
Tetracycline	Tetracycline	7	*tet(A)*	*pCFSAN059940*
Quinolones	Ciprofloxacin	7	*gyrA (p.D87Y)* ^b^	*–*
Nitrofurans	Nitrofurantoin	7	nfsA (p.W159^*^)^b^ nfsB (p.Q137^*^)^b^	*–*
Fosfomycin	Fosfomycin	0	*fosA3*	*pCFSAN059940*
Folate pathway inhibitor	Sulfamethoxazole ^+ ^ Trimethoprim	0	*sul1* *dfrA14*	*pCFSAN059940*
Macrolides	Azithromycin	0	–	–
Carbapenems	Ertapenem	0	–	–

^a^chromosomal-encoded gene.

^b^chromosomal-gene mutation (SNP).

### Clonal analysis

**Fig 2:**
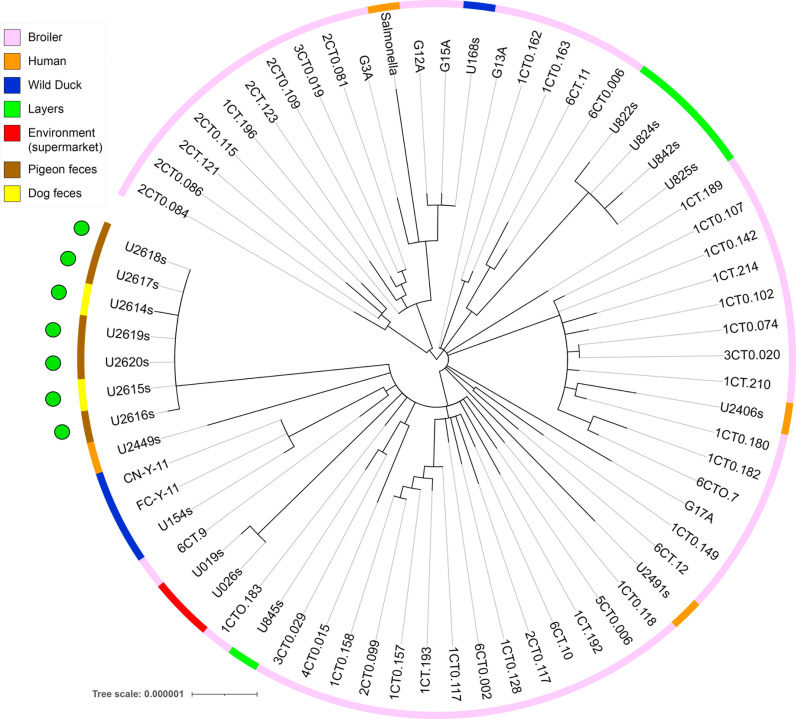
SNP tree analysis of *S.* Infantis isolates. Green circles show the strains of this study. All strains belonged to ST32.

**Fig 3:**
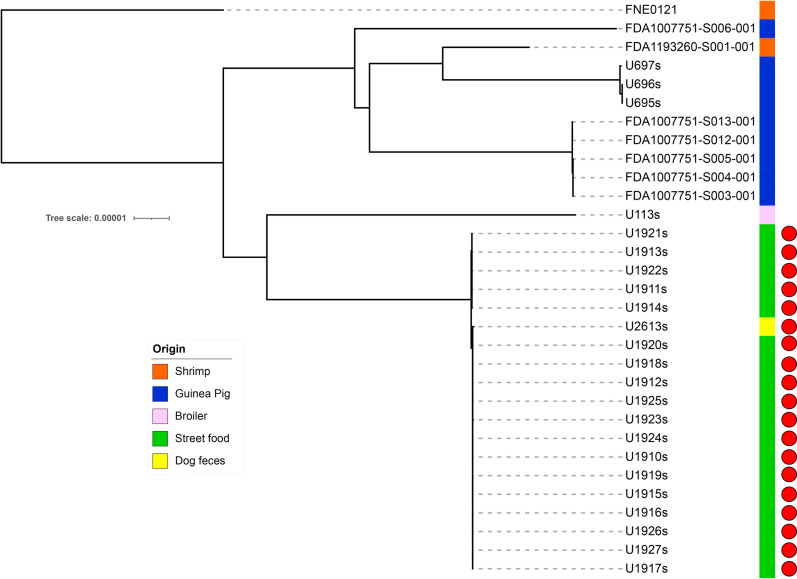
SNP tree analysis of *S.* Typhimurium isolates. Red circles show the strains of this study.

All *S.* Typhimurium strains from food belonged to ST19 and *S*. Infantis from feces to ST32 according to the Multi Locus Sequence Type (MLST) scheme. However, the Hierarchical clustering of cgMLST (HierCC)[29] allowed to differentiate 5 different cgST in *S.* Typhimurium and 5 cgST in *S.* Infantis at HC0 (cgMLST V2 +  HierCC V1) which are practically indistinguishable. Furthermore, from the HC2 level (cgMLST V2 +  HierCC V1), no difference between strains of each serotype could be identified (S3 file). These results show that strains of each serotype were highly clonal. These strains were not related to *Salmonella* genomes of other origins (Figs 2 and 3).

## Discussion

The present work is the first report of *Salmonella* in ready-to-eat street food in Ecuador. The presence of *Salmonella* isolated in this study reached 10%, which agrees with regional reports of *Salmonella-*contaminated street food in Colombia [30,31], Brazil [32], and other developing countries in Asia and Africa [33–35]. Although the diversity of food matrices and local behaviors make it difficult to perform comparisons between countries, it is important to highlight that this type of food is a potential source of foodborne disease-causing bacteria.

Given that most food samples had some thermal treatment, positive results could be related to cross-contamination events in the storage or distribution of foods. These kinds of events have been widely documented, mainly associating cookware with contaminated raw meat [36–38]. The presence of pathogens in street food might also be related to the hygienic status of vendors, serving practices, and the unavailability of running water [39,40]. Therefore, in situ, manipulation of raw ingredients and the usage of undercooked ingredients in final dishes (e.g.,: homemade salad and homemade mayonnaise) are risk factors for the presence of *Salmonella* in street foods [41–43]. This fact can be demonstrated in this study, where the presence of sauce or eggs increases up to 15 times the probability of *Salmonella* isolation independently of other ingredients or dressings.

On the other hand, urban animals such as dogs and pigeons could play a role in the epidemiology of *Salmonella* in highly populated cities, either due to their potential role as reservoirs or carriers of this pathogen [44,45]. Some studies have reported *S.* Infantis, *S.* Typhimurium, and other serotypes in urban fauna in North America, South America, Europe, and Asia [45–53]. In this study, *Salmonella* isolates of street food clustered with one isolate of dog feces origin. This fact shows the possible implication of contaminated street food with the presence of domestic animals in the environments where food is prepared. However, zooanthroponosis events (like the consumption of leftovers by dogs) could also explain this observation. These potential interactions highlight the necessity of addressing the epidemiology of *Salmonella* as a whole, to better control its transmission to humans [54]. In this context, emphasis should be placed on contributing factors of food cross-contamination including the hygienic practices implemented during the preparation of street foods and avoiding feral and domestic animals on the vicinities of food street vendors.

*S* Typhimurium and *S.* Infantis are the predominant serotypes in this study. These findings are of special relevance considering that these pathogens are among the main causal agents of gastroenteritis associated with contaminated food [55]. The analysis of clonality showed that isolates of both serotypes belonged to specific sequence types. *S*. Typhimurium isolates belonged to the ST19 which has been reported as a food contaminant in several studies [56–60]. In the case of *S*. Infantis, isolates of pigeon and dog origin clustered together showing the close relation that animals living in the same environment could have in terms of *Salmonella* colonization. These isolates belonged to the sequence type ST32 which has been extensively reported in Ecuador in the poultry production chain [21,61,62]. Although these strains did not cluster with other ST32 strains isolated on multiple origins in Ecuador, these results highlight the importance of urban fauna as reservoirs of *S*. Infantis and the implications that these animals could have in terms of public health [63]. *S.* Typhimurium did not show clonality with other sources; however, a cluster was found that includes a strain from dog feces, supporting the hypothesis of cross-contamination. This is important due to its role in the epidemiology of *Salmonella*, which includes the transmission of genetic determinants of antimicrobial resistance (AMR). Since this evidence does not show a relation of these strains as contaminants of sampled food, a larger sampling should be conducted to confirm this assumption.

Except for streptomycin, all *S*. Typhimurium were susceptible to tested antibiotics. These results differ from other regional reports in food and humans where this serotype usually presents multidrug-resistant patterns [64–68]. This finding is supported by clonality analysis, which demonstrated that the strains in this study are not closely related to strains of *S.* Typhimurium with higher resistance profiles from other origins. This suggests that the clones isolated in this study may be circulating specifically within this niche. However, more extensive sampling is required to confirm this hypothesis.

Multi-drug resistant *S*. Infantis in the region has been previously reported in animals, food, and the environment [62,69–71]. Furthermore, this serotype has been documented in broilers exhibiting extreme drug resistance, resistance to disinfectants, enhanced virulence, and improved ability to form biofilms. These characteristics have been linked to pESI-like plasmids [21,54,62]. Except for cefoxitin, amoxicillin +  clavulanic acid, sulfamethoxazole +  trimethoprim, and fosfomycin; the resistant phenotypes could be related to the genetic determinants of resistance present in the strains.

Extended-spectrum beta-lactamases (ESBLs) deactivate a broad array of beta-lactams, ranging from penicillin to third-generation cephalosporins. However, these enzymes typically do not affect beta-lactamase inhibitors and cephamycins [72]. Historically, resistance to these antibiotics has been primarily linked to AmpC beta-lactamases (class C or group 1) [73]. In the strains examined, *bla*_CTX-M-65_ was the only beta-lactamase gene detected, suggesting that the spectrum of activity for this particular allele does not extend to cefoxitin and amoxicillin +  clavulanic acid. This outcome aligns with previous findings in genetically similar strains, supporting this hypothesis [74,75]. Nevertheless, additional experimental verification is required to confirm these results.

Fosfomycin has emerged as a primary treatment option for urinary tract infections and a last-resort therapy for severe infections caused by Gram-negative bacteria [76,77]. Although the present study does not integrate a transcriptional analysis, it was notable that all fosfomycin-susceptible isolates in this study exhibited a lack of *fosA3* gene expression. Although such observations are scarce, similar findings have been reported in other bacteria [78,79]. It should be noted that most research concentrates on identifying genetic markers in resistant phenotypes [80,81], which may lead to an underestimation of the presence of *fosA3* in susceptible isolates. We considered the possibility of a non-functional product arising from an incomplete or modified gene. However, fosA3 genes showed 100% coverage and identity in all cases (data not shown). Therefore, this event might be attributed to the genetic context of the strains and the absence of promoter regions necessary for *fosA3* gene expression (not functional gene or subexpression)[82]. A comparable situation occurs with the folate pathway inhibitors, where despite the presence of resistance genes to trimethoprim (*dfrA14*) and sulfamethoxazole (*sul1*), no phenotypic resistance to the combined action of these antibiotics was observed. It is noteworthy that even though laboratory conditions have been proposed as a source of bias when analyzing antimicrobial resistance, the phenomenon of silent genes has been observed to be a common event [83]. Given these facts, further research is essential to fully understand these dynamics in future research.

The study of plasmids structure, epidemiology, and expression is vital for pathogen surveillance. Historically, plasmids were categorized by Incompatibility groups (Inc groups), which describe their coexistence in cells but do not reflect genetic variants or phylogenetic relationships [84]. The introduction of plasmid multilocus sequence typing (pMLST) in 2014 provides a more precise analysis of plasmid diversity and evolution by examining specific loci [24]. Combining Inc groups with pMLST enhances our understanding of plasmid dynamics in bacterial populations. In our study, no resistance genes could be found in plasmids of *S.* Typhimurium. These plasmids have the replicons IncFIB(S) and IncFII(S) which belong to the Incompatibility Group F (IncF). Additionally, these plasmids belonged to the pMLST [S1:A-:B17]. Comparable results were reported in isolates of this serotype with similar features from pigs in Denmark [85]. However, this pMLST was also reported in *S.* Typhimurium from humans with the *bla*_TEM-1B_ gene harbored in plasmids with different replicons (FIB(S), FII(S))[86]. It must be noted that the pMLST [S1:A-:B17] has not been reported in other bacteria than *S*. Typhimurium which could indicate that these plasmids are highly conserved in this *Salmonella* serotype.

On the other hand, the plasmid in *S*. Infantis strains of this study (pCFSAN059940-like) belongs to the Incompatibility Group I1 (IncI1) with the replicon IncFIB(pN55391). This plasmid does not have an assigned pMLST, however, it is highly similar to the pMLST 71. It should be noted that plasmids of similar characteristics (the same replicon and pMLST) have been previously reported in South America [21,62,69,87]. On the other hand, other plasmids with this replicon and also lack a known *repI1* sequence could be identified in Europe but show a close genetic structure to the pMLST 209 [88,89]. These plasmids, generically called pESI or pESI-like, are related to poultry worldwide and associated with MDR genes. Additionally, this Inc group contains several plasmids reported in *Salmonella* and *E. coli* of human and animal origin [90].

These findings indicate the potential presence of plasmids, identified by unique genetic markers, that could have increased affinity to particular bacteria or serotypes. Furthermore, the observation that *Salmonella* strains of the same serotype carried plasmids with identical pMLST supports the conclusions drawn from the previous clonality analysis. Such insights could be valuable for integration into surveillance and control strategies. Besides, the combination of replicon identities and pMLST typing gives an interesting way to overcome the challenges that the genomic plasticity of plasmids represents when understanding the epidemiological dynamics of *Salmonella*.

The study acknowledges certain factors that may influence the findings, such as the absence of a single protocol, which limited direct comparison between food and animal feces, and the limited sample size included in the analysis. Nevertheless, this study marks the initial scientific documentation of *Salmonella* in Ecuadorian street food, underscoring the interconnected roles of urban environments and fauna. It raises critical questions regarding the direction of transmission: Are the street foods contaminated due to the presence of dogs, or are dogs contracting *Salmonella* from contaminated street food? This highlights the need for a One Health approach to fully understand and address the transmission dynamics in urban settings. Additionally, the study emphasizes the importance of plasmid analysis for understanding bacterial evolution and underscores the need for comprehensive food safety and animal management strategies.

## Supporting information

S1 File Sampling site coordinates. Photographs and coordinates of exact location of Quito and the Carolina Park are presented in the S1 file.
(PDF)

S2 File Food and animal samples description and location. Core components of food, animal source of samples and location are listed in S2 file.
(XLSX)

S3 File Genotypic and phenotypic data of the *Salmonella* isolates. Accession numbers of all genomes studied genes, and their metadata as well as the phenotypic antimicrobial responses are listed in the S3 file.
(XLSX)
