# Supplementary material for: Salmonella isolated from street foods and environment of an urban park: A whole genome sequencing approach
Source: PLoS One. 2025 Apr 2;20(4):e0320735. doi: 10.1371/journal.pone.0320735 (PMC11964277; doi:10.1371/journal.pone.0320735)

Sampling site coordinates

Ecuador (country) -1°47'34.68" S -78°08'12.80" W

Pichincha (province) 0°04'59.99" N -78°30'0.00" W

Quito (City) 0°13'0.01" N -78°30'0.00" W

Carolina Park, 0º11’019” S -78º29’04.9” W

GMT: -5h. Altitude: 2762 m. Address: Avenida de los Shyris, La Carolina, Quito, Pichincha, 170502, Ecuador.

Photographs

Carolina Park Photographs by Rommy Terán

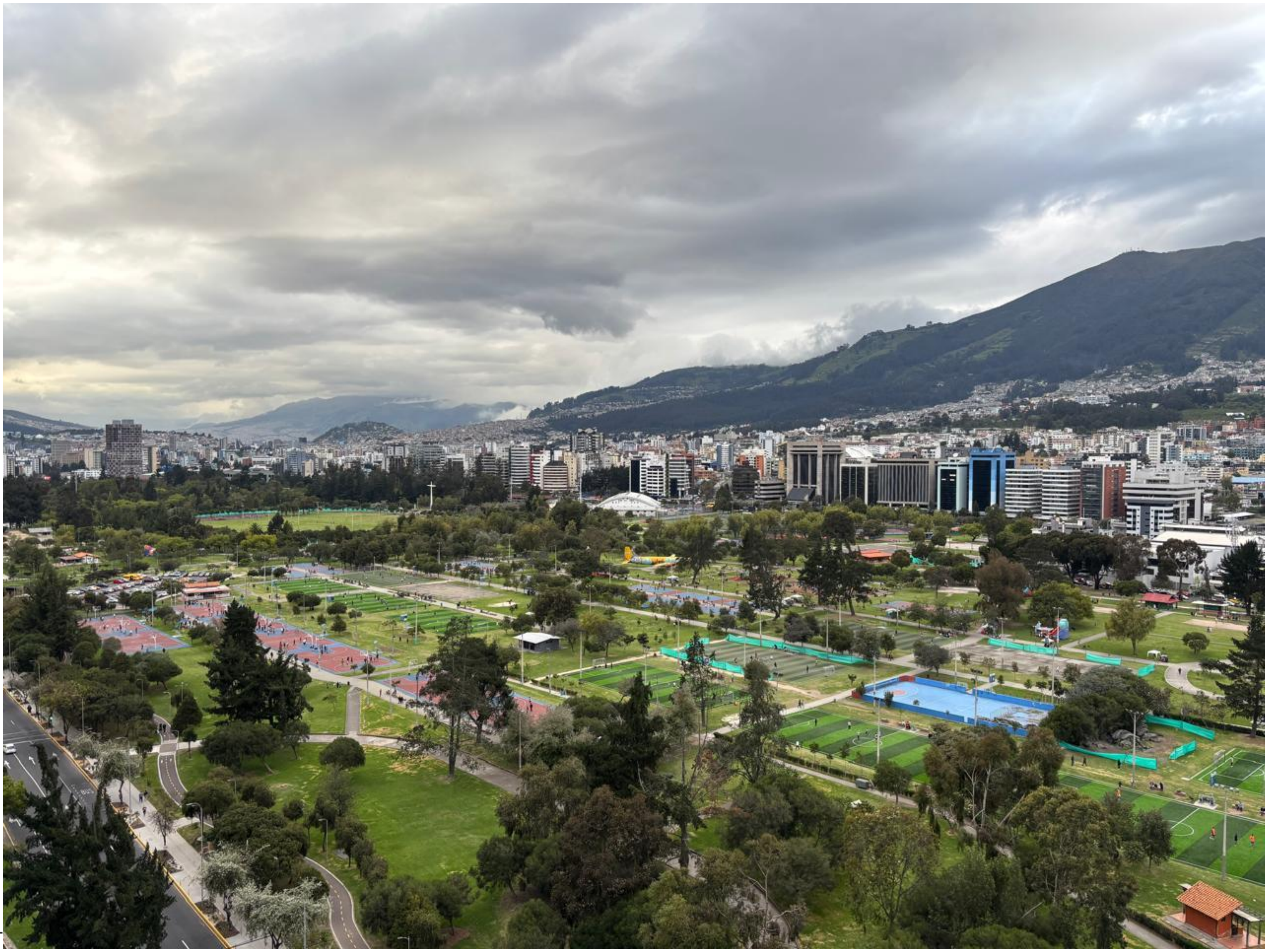

T

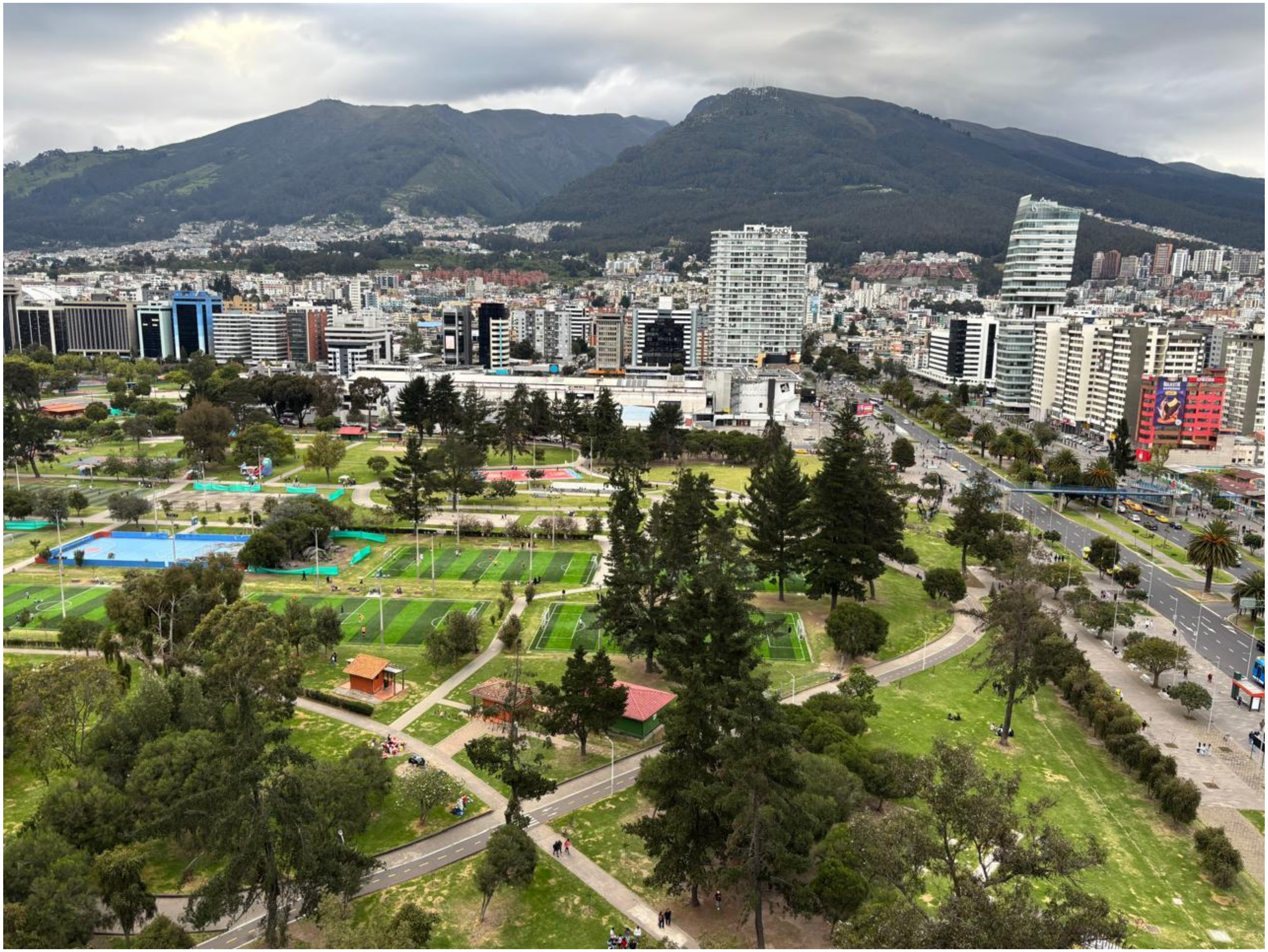

Supplement: S1 File — Photographs and coordinates of exact location of Quito and the Carolina Park are presented in the S1 file. (PDF) [file pone.0320735.s001.pdf]
